# Early adolescents’ sexual and reproductive health literacy in Lao PDR

**DOI:** 10.1093/eurpub/ckac130.039

**Published:** 2022-10-25

**Authors:** HK Lee, YS Yang, SK Kim, V Vongxay, YM Lee

**Affiliations:** 1 Nursing, Yonsei University, Seoul, South Korea; 2 Regional Office, Korea International Cooperation Agency, Vientiane, Laos; 3 Nursing, Soonchunhyang, Cheonan, South Korea; 4 Public Health, University of Health Sciences, Vientiane, Laos; 5 Nursing, DePaul University, Chicago, USA

## Abstract

**Background:**

Sexual and reproductive health literacy (SRHL) refers to the ability to access, understand, appraise, and apply information for decision-making related to sexual and reproductive health. The low level of SRHL in adolescents increases their sexually risky behaviors and endangers sexual health. Although early adolescence is a critical development period for forming initial views on sexuality and is often a time for attempting risky behaviors, studies on SRHL for early adolescents are fairly limited in Las PDR. As an initial step for the development of a global health project between Lao PDR and South Korea, this study assessed the level of SRHL and the differences in gender among early adolescents in Lao PDR.

**Methods:**

Participants were 235 students conveniently recruited from one junior high school each in two provinces in Lao PDR. SRHL was measured using the 39-item Teen Pregnancy Health Literacy scale consisting of 4 subscales of finding, understanding, appraisal, and application. The scores were classified into inadequate, problematic, sufficient, and excellent using the SRHL index formula. The mean differences in gender were compared using t-test.

**Results:**

The mean of the SRHL scores of the participants was 19.07 (±10.57). The mean score was significantly lower for girls, at 17.67 (±11.22) than for boys, at 21.37 (±9.05) (p = .006). Significant differences were further identified in all four sub-domains of SRHL: finding (p = .025), understanding (p = .005), appraisal (p = .041), and application (p = .029). The majority of participants (91.7%) were categorized as having an ‘inadequate’ or ‘problematic’ level of SRHL.

**Conclusions:**

The level of SRHL among most early adolescents was found to be inadequate. The level of SRHL among girls was much lower than that among boys. The findings suggest a gender-specific approach to developing health education programs to improve SRHL among early adolescents and prevent future sexually risky behaviors in Lao PDR.

**Key messages:**

- Most of the early adolescents who participated in this study were categorized as having an inadequate or problematic level of SRHL.
- This study suggests a gender-specific approach to develop and implement interventions to improve adolescents’ SRHL, particularly in lower-middle-income countries, to ultimately enhance sexual health.

